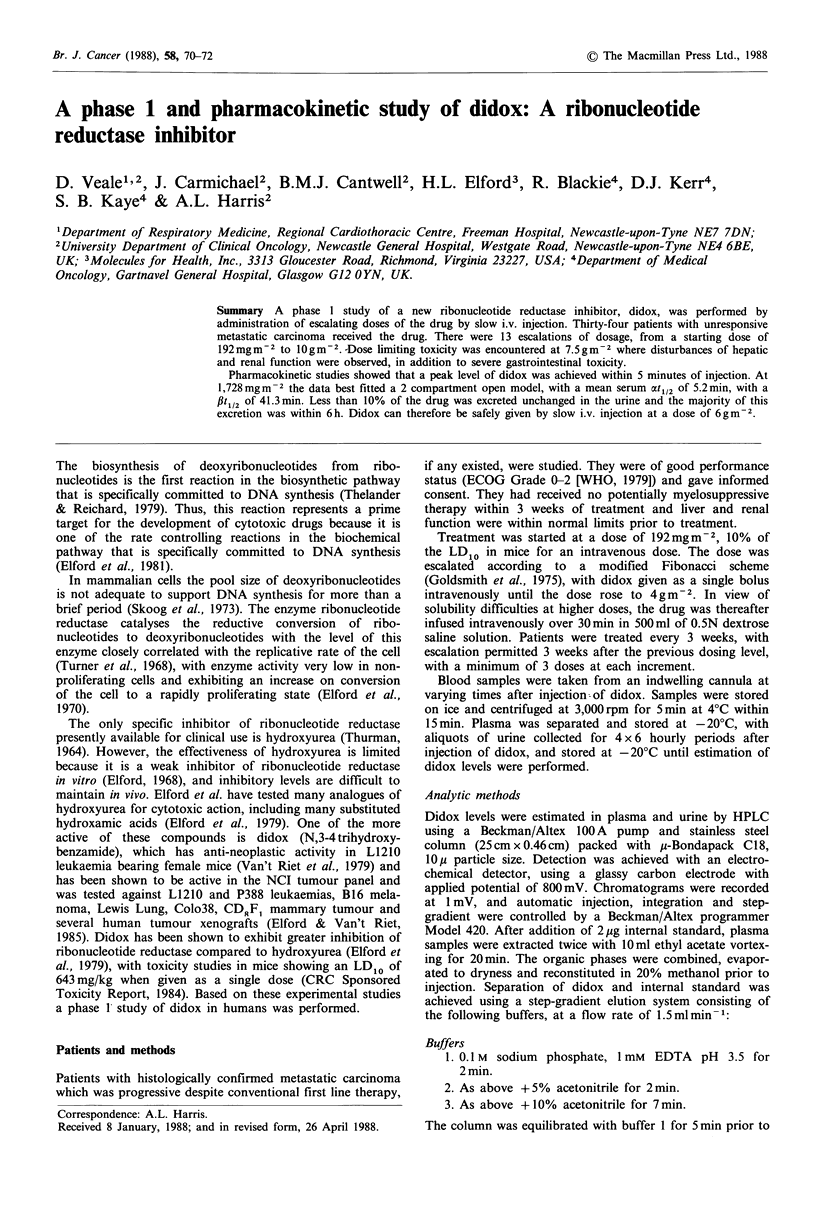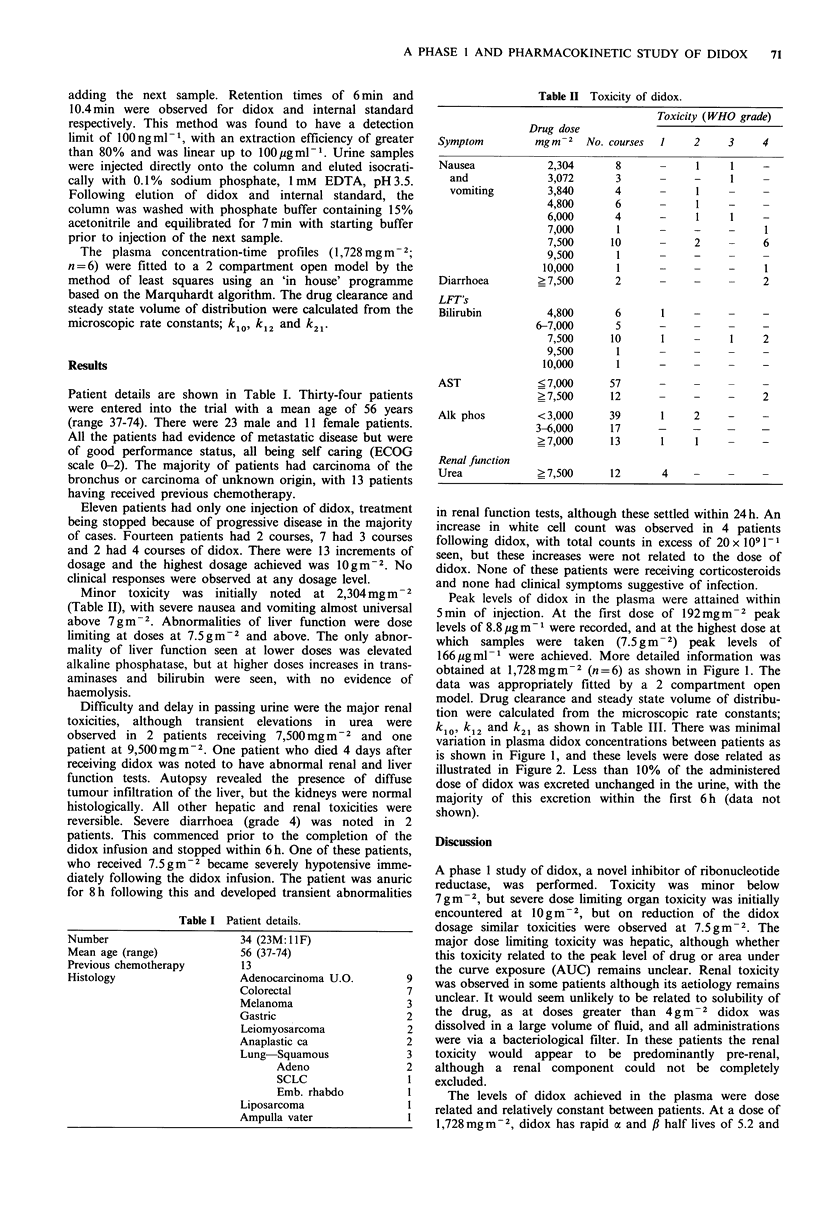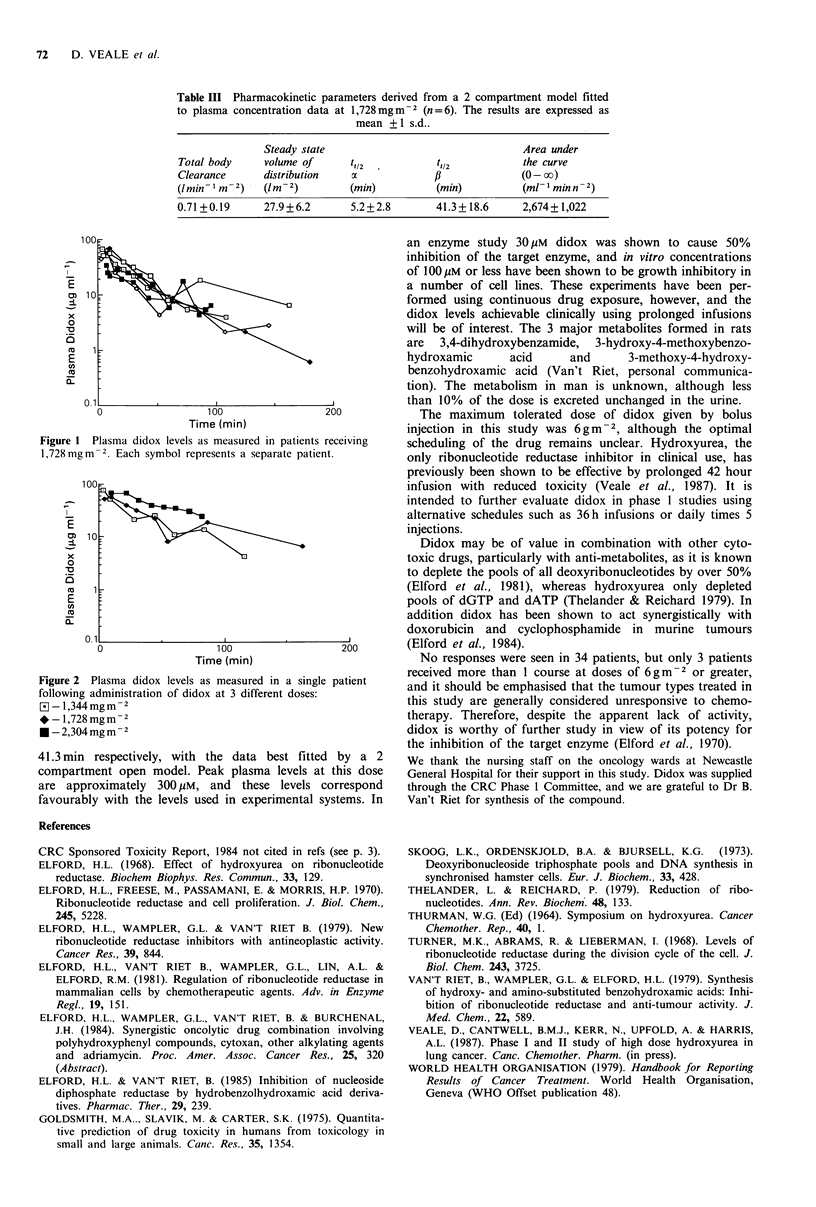# A phase 1 and pharmacokinetic study of didox: a ribonucleotide reductase inhibitor.

**DOI:** 10.1038/bjc.1988.164

**Published:** 1988-07

**Authors:** D. Veale, J. Carmichael, B. M. Cantwell, H. L. Elford, R. Blackie, D. J. Kerr, S. B. Kaye, A. L. Harris

**Affiliations:** Department of Respiratory Medicine, Freeman Hospital, Newcastle-upon-Tyne, UK.

## Abstract

A phase 1 study of a new ribonucleotide reductase inhibitor, didox, was performed by administration of escalating doses of the drug by slow i.v. injection. Thirty-four patients with unresponsive metastatic carcinoma received the drug. There were 13 escalations of dosage, from a starting dose of 192 mg m-2 to 10 g m-2. Dose limiting toxicity was encountered at 7.5 g m-2 where disturbances of hepatic and renal function were observed, in addition to severe gastrointestinal toxicity. Pharmacokinetic studies showed that a peak level of didox was achieved within 5 minutes of injection. At 1,728 mg m-2 the data best fitted a 2 compartment open model, with a mean serum alpha t1/2 of 5.2 min, with a beta t1/2 of 41.3 min. Less than 10% of the drug was excreted unchanged in the urine and the majority of this excretion was within 6 h. Didox can therefore be safely given by slow i.v. injection at a dose of 6 g m-2.


*Br. J. Cancer* (1988), **58**, 70–72 © The Macmillan Press Ltd., 1988

### A phase 1 and pharmacokinetic study of didox: A ribonucleotide reductase inhibitor

D. Veale[1,2], J. Carmichael[2], B.M.J. Cantwell[2], H.L. Elford[3], R. Blackie[4], D.J. Kerr[4], S. B. Kaye[4] & A.L. Harris[2]

[1]*Department of Respiratory Medicine, Regional Cardiothoracic Centre, Freeman Hospital, Newcastle-upon-Tyne NE7 7DN;* [2]*University Department of Clinical Oncology, Newcastle General Hospital, Westgate Road, Newcastle-upon-Tyne NE4 6BE, UK;* [3]*Molecules for Health, Inc., 3313 Gloucester Road, Richmond, Virginia 23227, USA;* [4]*Department of Medical Oncology, Gartnavel General Hospital, Glasgow G12 0YN, UK.*

**Summary** A phase 1 study of a new ribonucleotide reductase inhibitor, didox, was performed by administration of escalating doses of the drug by slow i.v. injection. Thirty-four patients with unresponsive metastatic carcinoma received the drug. There were 13 escalations of dosage, from a starting dose of 192 mg m$^{-2}$ to 10 g m$^{-2}$. Dose limiting toxicity was encountered at 7.5 g m$^{-2}$ where disturbances of hepatic and renal function were observed, in addition to severe gastrointestinal toxicity.

Pharmacokinetic studies showed that a peak level of didox was achieved within 5 minutes of injection. At 1,728 mg m$^{-2}$ the data best fitted a 2 compartment open model, with a mean serum $\alpha t_{1/2}$ of 5.2 min, with a $\beta t_{1/2}$ of 41.3 min. Less than 10% of the drug was excreted unchanged in the urine and the majority of this excretion was within 6 h. Didox can therefore be safely given by slow i.v. injection at a dose of 6 g m$^{-2}$.

The biosynthesis of deoxyribonucleotides from ribonucleotides is the first reaction in the biosynthetic pathway that is specifically committed to DNA synthesis (Thelander & Reichard, 1979). Thus, this reaction represents a prime target for the development of cytotoxic drugs because it is one of the rate controlling reactions in the biochemical pathway that is specifically committed to DNA synthesis (Elford *et al.*, 1981).

In mammalian cells the pool size of deoxyribonucleotides is not adequate to support DNA synthesis for more than a brief period (Skoog *et al.*, 1973). The enzyme ribonucleotide reductase catalyses the reductive conversion of ribonucleotides to deoxyribonucleotides with the level of this enzyme closely correlated with the replicative rate of the cell (Turner *et al.*, 1968), with enzyme activity very low in non-proliferating cells and exhibiting an increase on conversion of the cell to a rapidly proliferating state (Elford *et al.*, 1970).

The only specific inhibitor of ribonucleotide reductase presently available for clinical use is hydroxyurea (Thurman, 1964). However, the effectiveness of hydroxyurea is limited because it is a weak inhibitor of ribonucleotide reductase *in vitro* (Elford, 1968), and inhibitory levels are difficult to maintain *in vivo*. Elford *et al.* have tested many analogues of hydroxyurea for cytotoxic action, including many substituted hydroxamic acids (Elford *et al.*, 1979). One of the more active of these compounds is didox (N,3-4 trihydroxybenzamide), which has anti-neoplastic activity in L1210 leukaemia bearing female mice (Van't Riet *et al.*, 1979) and has been shown to be active in the NCI tumour panel and was tested against L1210 and P388 leukaemias, B16 melanoma, Lewis Lung, Colo38, $CD_8F_1$ mammary tumour and several human tumour xenografts (Elford & Van't Riet, 1985). Didox has been shown to exhibit greater inhibition of ribonucleotide reductase compared to hydroxyurea (Elford *et al.*, 1979), with toxicity studies in mice showing an $LD_{10}$ of 643 mg/kg when given as a single dose (CRC Sponsored Toxicity Report, 1984). Based on these experimental studies a phase 1 study of didox in humans was performed.

### Patients and methods

Patients with histologically confirmed metastatic carcinoma which was progressive despite conventional first line therapy, if any existed, were studied. They were of good performance status (ECOG Grade 0–2 [WHO, 1979]) and gave informed consent. They had received no potentially myelosuppressive therapy within 3 weeks of treatment and liver and renal function were within normal limits prior to treatment.

Treatment was started at a dose of 192 mg m$^{-2}$, 10% of the $LD_{10}$ in mice for an intravenous dose. The dose was escalated according to a modified Fibonacci scheme (Goldsmith *et al.*, 1975), with didox given as a single bolus intravenously until the dose rose to 4 g m$^{-2}$. In view of solubility difficulties at higher doses, the drug was thereafter infused intravenously over 30 min in 500 ml of 0.5N dextrose saline solution. Patients were treated every 3 weeks, with escalation permitted 3 weeks after the previous dosing level, with a minimum of 3 doses at each increment.

Blood samples were taken from an indwelling cannula at varying times after injection of didox. Samples were stored on ice and centrifuged at 3,000 rpm for 5 min at 4°C within 15 min. Plasma was separated and stored at −20°C, with aliquots of urine collected for 4 x 6 hourly periods after injection of didox, and stored at −20°C until estimation of didox levels were performed.

### *Analytic methods*

Didox levels were estimated in plasma and urine by HPLC using a Beckman/Altex 100A pump and stainless steel column (25 cm x 0.46 cm) packed with $\mu$-Bondapack C18, 10 $\mu$ particle size. Detection was achieved with an electrochemical detector, using a glassy carbon electrode with applied potential of 800 mV. Chromatograms were recorded at 1 mV, and automatic injection, integration and step-gradient were controlled by a Beckman/Altex programmer Model 420. After addition of 2 $\mu$g internal standard, plasma samples were extracted twice with 10 ml ethyl acetate vortexing for 20 min. The organic phases were combined, evaporated to dryness and reconstituted in 20% methanol prior to injection. Separation of didox and internal standard was achieved using a step-gradient elution system consisting of the following buffers, at a flow rate of 1.5 ml min$^{-1}$:

*Buffers*

1. 0.1 M sodium phosphate, 1 mM EDTA pH 3.5 for 2 min.
2. As above +5% acetonitrile for 2 min.
3. As above +10% acetonitrile for 7 min.

The column was equilibrated with buffer 1 for 5 min prior to

Correspondence: A.L. Harris.
Received 8 January, 1988; and in revised form, 26 April 1988.

adding the next sample. Retention times of 6 min and 10.4 min were observed for didox and internal standard respectively. This method was found to have a detection limit of $100\,\mathrm{ng\,ml^{-1}}$, with an extraction efficiency of greater than 80% and was linear up to $100\,\mu\mathrm{g\,ml^{-1}}$. Urine samples were injected directly onto the column and eluted isocratically with 0.1% sodium phosphate, 1 mM EDTA, pH 3.5. Following elution of didox and internal standard, the column was washed with phosphate buffer containing 15% acetonitrile and equilibrated for 7 min with starting buffer prior to injection of the next sample.

The plasma concentration-time profiles ($1{,}728\,\mathrm{mg\,m^{-2}}$; $n=6$) were fitted to a 2 compartment open model by the method of least squares using an 'in house' programme based on the Marquhardt algorithm. The drug clearance and steady state volume of distribution were calculated from the microscopic rate constants; $k_{10}$, $k_{12}$ and $k_{21}$.

### Results

Patient details are shown in Table I. Thirty-four patients were entered into the trial with a mean age of 56 years (range 37-74). There were 23 male and 11 female patients. All the patients had evidence of metastatic disease but were of good performance status, all being self caring (ECOG scale 0–2). The majority of patients had carcinoma of the bronchus or carcinoma of unknown origin, with 13 patients having received previous chemotherapy.

Eleven patients had only one injection of didox, treatment being stopped because of progressive disease in the majority of cases. Fourteen patients had 2 courses, 7 had 3 courses and 2 had 4 courses of didox. There were 13 increments of dosage and the highest dosage achieved was $10\,\mathrm{g\,m^{-2}}$. No clinical responses were observed at any dosage level.

Minor toxicity was initially noted at $2{,}304\,\mathrm{mg\,m^{-2}}$ (Table II), with severe nausea and vomiting almost universal above $7\,\mathrm{g\,m^{-2}}$. Abnormalities of liver function were dose limiting at doses at $7.5\,\mathrm{g\,m^{-2}}$ and above. The only abnormality of liver function seen at lower doses was elevated alkaline phosphatase, but at higher doses increases in transaminases and bilirubin were seen, with no evidence of haemolysis.

Difficulty and delay in passing urine were the major renal toxicities, although transient elevations in urea were observed in 2 patients receiving $7{,}500\,\mathrm{mg\,m^{-2}}$ and one patient at $9{,}500\,\mathrm{mg\,m^{-2}}$. One patient who died 4 days after receiving didox was noted to have abnormal renal and liver function tests. Autopsy revealed the presence of diffuse tumour infiltration of the liver, but the kidneys were normal histologically. All other hepatic and renal toxicities were reversible. Severe diarrhoea (grade 4) was noted in 2 patients. This commenced prior to the completion of the didox infusion and stopped within 6 h. One of these patients, who received $7.5\,\mathrm{g\,m^{-2}}$ became severely hypotensive immediately following the didox infusion. The patient was anuric for 8 h following this and developed transient abnormalities in renal function tests, although these settled within 24 h. An increase in white cell count was observed in 4 patients following didox, with total counts in excess of $20\times10^{9}\,\mathrm{l^{-1}}$ seen, but these increases were not related to the dose of didox. None of these patients were receiving corticosteroids and none had clinical symptoms suggestive of infection.

Peak levels of didox in the plasma were attained within 5 min of injection. At the first dose of $192\,\mathrm{mg\,m^{-2}}$ peak levels of $8.8\,\mu\mathrm{g\,m^{-1}}$ were recorded, and at the highest dose at which samples were taken ($7.5\,\mathrm{g\,m^{-2}}$) peak levels of $166\,\mu\mathrm{g\,ml^{-1}}$ were achieved. More detailed information was obtained at $1{,}728\,\mathrm{mg\,m^{-2}}$ ($n=6$) as shown in Figure 1. The data was appropriately fitted by a 2 compartment open model. Drug clearance and steady state volume of distribution were calculated from the microscopic rate constants; $k_{10}$, $k_{12}$ and $k_{21}$ as shown in Table III. There was minimal variation in plasma didox concentrations between patients as is shown in Figure 1, and these levels were dose related as illustrated in Figure 2. Less than 10% of the administered dose of didox was excreted unchanged in the urine, with the majority of this excretion within the first 6 h (data not shown).

**Table I** Patient details.

| | | |
|---|---|---|
| Number | 34 (23M:11F) | |
| Mean age (range) | 56 (37-74) | |
| Previous chemotherapy | 13 | |
| Histology | Adenocarcinoma U.O. | 9 |
| | Colorectal | 7 |
| | Melanoma | 3 |
| | Gastric | 2 |
| | Leiomyosarcoma | 2 |
| | Anaplastic ca | 2 |
| | Lung—Squamous | 3 |
| | Adeno | 2 |
| | SCLC | 1 |
| | Emb. rhabdo | 1 |
| | Liposarcoma | 1 |
| | Ampulla vater | 1 |

**Table II** Toxicity of didox.

| *Symptom* | *Drug dose* $mg\,m^{-2}$ | *No. courses* | *Toxicity (WHO grade)* 1 | 2 | 3 | 4 |
|---|---|---|---|---|---|---|
| Nausea and vomiting | 2,304 | 8 | – | 1 | 1 | – |
| | 3,072 | 3 | – | – | 1 | – |
| | 3,840 | 4 | – | 1 | – | – |
| | 4,800 | 6 | – | 1 | – | – |
| | 6,000 | 4 | – | 1 | 1 | – |
| | 7,000 | 1 | – | – | – | 1 |
| | 7,500 | 10 | – | 2 | – | 6 |
| | 9,500 | 1 | – | – | – | – |
| | 10,000 | 1 | – | – | – | 1 |
| Diarrhoea | ≧7,500 | 2 | – | – | – | 2 |
| *LFT's* | | | | | | |
| Bilirubin | 4,800 | 6 | 1 | – | – | – |
| | 6–7,000 | 5 | – | – | – | – |
| | 7,500 | 10 | 1 | – | 1 | 2 |
| | 9,500 | 1 | – | – | – | – |
| | 10,000 | 1 | – | – | – | – |
| AST | ≦7,000 | 57 | – | – | – | – |
| | ≧7,500 | 12 | – | – | – | 2 |
| Alk phos | <3,000 | 39 | 1 | 2 | – | – |
| | 3–6,000 | 17 | – | – | – | – |
| | ≧7,000 | 13 | 1 | 1 | – | – |
| *Renal function* | | | | | | |
| Urea | ≧7,500 | 12 | 4 | – | – | – |

### Discussion

A phase 1 study of didox, a novel inhibitor of ribonucleotide reductase, was performed. Toxicity was minor below $7\,\mathrm{g\,m^{-2}}$, but severe dose limiting organ toxicity was initially encountered at $10\,\mathrm{g\,m^{-2}}$, but on reduction of the didox dosage similar toxicities were observed at $7.5\,\mathrm{g\,m^{-2}}$. The major dose limiting toxicity was hepatic, although whether this toxicity related to the peak level of drug or area under the curve exposure (AUC) remains unclear. Renal toxicity was observed in some patients although its aetiology remains unclear. It would seem unlikely to be related to solubility of the drug, as at doses greater than $4\,\mathrm{g\,m^{-2}}$ didox was dissolved in a large volume of fluid, and all administrations were via a bacteriological filter. In these patients the renal toxicity would appear to be predominantly pre-renal, although a renal component could not be completely excluded.

The levels of didox achieved in the plasma were dose related and relatively constant between patients. At a dose of $1{,}728\,\mathrm{mg\,m^{-2}}$, didox has rapid $\alpha$ and $\beta$ half lives of 5.2 and

**Table III** Pharmacokinetic parameters derived from a 2 compartment model fitted to plasma concentration data at 1,728 mg m$^{-2}$ ($n$=6). The results are expressed as mean ±1 s.d..

| *Total body Clearance* ($l\,min^{-1}\,m^{-2}$) | *Steady state volume of distribution* ($l\,m^{-2}$) | $t_{1/2}$ α (*min*) | $t_{1/2}$ β (*min*) | *Area under the curve* (0−∞) ($ml^{-1}\,min\,n^{-2}$) |
|---|---|---|---|---|
| 0.71±0.19 | 27.9±6.2 | 5.2±2.8 | 41.3±18.6 | 2,674±1,022 |

**Figure 1** Plasma didox levels as measured in patients receiving 1,728 mg m$^{-2}$. Each symbol represents a separate patient.

**Figure 2** Plasma didox levels as measured in a single patient following administration of didox at 3 different doses:
▣ – 1,344 mg m$^{-2}$
◆ – 1,728 mg m$^{-2}$
■ – 2,304 mg m$^{-2}$

41.3 min respectively, with the data best fitted by a 2 compartment open model. Peak plasma levels at this dose are approximately 300 μM, and these levels correspond favourably with the levels used in experimental systems. In an enzyme study 30 μM didox was shown to cause 50% inhibition of the target enzyme, and *in vitro* concentrations of 100 μM or less have been shown to be growth inhibitory in a number of cell lines. These experiments have been performed using continuous drug exposure, however, and the didox levels achievable clinically using prolonged infusions will be of interest. The 3 major metabolites formed in rats are 3,4-dihydroxybenzamide, 3-hydroxy-4-methoxybenzohydroxamic acid and 3-methoxy-4-hydroxybenzohydroxamic acid (Van't Riet, personal communication). The metabolism in man is unknown, although less than 10% of the dose is excreted unchanged in the urine.

The maximum tolerated dose of didox given by bolus injection in this study was 6 g m$^{-2}$, although the optimal scheduling of the drug remains unclear. Hydroxyurea, the only ribonucleotide reductase inhibitor in clinical use, has previously been shown to be effective by prolonged 42 hour infusion with reduced toxicity (Veale *et al.*, 1987). It is intended to further evaluate didox in phase 1 studies using alternative schedules such as 36 h infusions or daily times 5 injections.

Didox may be of value in combination with other cytotoxic drugs, particularly with anti-metabolites, as it is known to deplete the pools of all deoxyribonucleotides by over 50% (Elford *et al.*, 1981), whereas hydroxyurea only depleted pools of dGTP and dATP (Thelander & Reichard 1979). In addition didox has been shown to act synergistically with doxorubicin and cyclophosphamide in murine tumours (Elford *et al.*, 1984).

No responses were seen in 34 patients, but only 3 patients received more than 1 course at doses of 6 g m$^{-2}$ or greater, and it should be emphasised that the tumour types treated in this study are generally considered unresponsive to chemotherapy. Therefore, despite the apparent lack of activity, didox is worthy of further study in view of its potency for the inhibition of the target enzyme (Elford *et al.*, 1970).

We thank the nursing staff on the oncology wards at Newcastle General Hospital for their support in this study. Didox was supplied through the CRC Phase 1 Committee, and we are grateful to Dr B. Van't Riet for synthesis of the compound.

### References

CRC Sponsored Toxicity Report, 1984 not cited in refs (see p. 3).

ELFORD, H.L. (1968). Effect of hydroxyurea on ribonucleotide reductase. *Biochem Biophys. Res. Commun.*, **33**, 129.

ELFORD, H.L., FREESE, M., PASSAMANI, E. & MORRIS, H.P. 1970). Ribonucleotide reductase and cell proliferation. *J. Biol. Chem.*, **245**, 5228.

ELFORD, H.L., WAMPLER, G.L. & VAN'T RIET B. (1979). New ribonucleotide reductase inhibitors with antineoplastic activity. *Cancer Res.*, **39**, 844.

ELFORD, H.L., VAN'T RIET B., WAMPLER, G.L., LIN, A.L. & ELFORD, R.M. (1981). Regulation of ribonucleotide reductase in mammalian cells by chemotherapeutic agents. *Adv. in Enzyme Regl.*, **19**, 151.

ELFORD, H.L., WAMPLER, G.L., VAN'T RIET, B. & BURCHENAL, J.H. (1984). Synergistic oncolytic drug combination involving polyhydroxyphenyl compounds, cytoxan, other alkylating agents and adriamycin. *Proc. Amer. Assoc. Cancer Res.*, **25**, 320 (*Abstract*).

ELFORD, H.L. & VAN'T RIET, B. (1985) Inhibition of nucleoside diphosphate reductase by hydrobenzolhydroxamic acid derivatives. *Pharmac. Ther.*, **29**, 239.

GOLDSMITH, M.A.., SLAVIK, M. & CARTER, S.K. (1975). Quantitative prediction of drug toxicity in humans from toxicology in small and large animals. *Canc. Res.*, **35**, 1354.

SKOOG, L.K., ORDENSKJOLD, B.A. & BJURSELL, K.G. (1973). Deoxyribonucleoside triphosphate pools and DNA synthesis in synchronised hamster cells. *Eur. J. Biochem.*, **33**, 428.

THELANDER, L. & REICHARD, P. (1979). Reduction of ribonucleotides. *Ann. Rev. Biochem.* **48**, 133.

THURMAN, W.G. (Ed) (1964). Symposium on hydroxyurea. *Cancer Chemother. Rep.*, **40**, 1.

TURNER, M.K., ABRAMS, R. & LIEBERMAN, I. (1968). Levels of ribonucleotide reductase during the division cycle of the cell. *J. Biol. Chem.* **243**, 3725.

VAN'T RIET, B., WAMPLER, G.L. & ELFORD, H.L. (1979). Synthesis of hydroxy- and amino-substituted benzohydroxamic acids: Inhibition of ribonucleotide reductase and anti-tumour activity. *J. Med. Chem.*, **22**, 589.

VEALE, D., CANTWELL, B.M.J., KERR, N., UPFOLD, A. & HARRIS, A.L. (1987). Phase I and II study of high dose hydroxyurea in lung cancer. *Canc. Chemother. Pharm.* (in press).

WORLD HEALTH ORGANISATION (1979). *Handbook for Reporting Results of Cancer Treatment*. World Health Organisation, Geneva (WHO Offset publication 48).